# Eyelid twitching in MuSK-myasthenia gravis

**DOI:** 10.1007/s13760-025-02811-1

**Published:** 2025-06-18

**Authors:** Vasiliki Zouvelou, Dimitra Tzavella, Thomas Zambelis

**Affiliations:** 1 1st Neurology Department, Eginition Hospital, National and Kapodistrian University of Athens, ERN EURO-MND, 72-74 Vas. Sophias Ave., 11528 Athens, Greece; 2 Henry Dunant Hospital, Athens, Greece

**Keywords:** Myasthenia Gravis, MuSK, Twiching, Eyelid

## Abstract

A 22-year-old female suffering from MuSK-myasthenia gravis presented with fatigable eyelid ptosis, fluctuated bulbar symptoms and bilateral eyelid twichings which were greatly reinforced after tight closure of eyes and contraction of orbicularis oculi muscles. The patient never received acetylcholinesterase inhibitor treatment. Persistent twichings, irrespectively of clinical status, were signs of cholinergic hyperactivity. MuSK antibodies disrupt MuSK-collagen Q interaction with resultant deficient binding of acetylcholinesterase at synaptic cleft and impaired breakdown of acetylcholine. In myasthenia gravis, clinical signs of cholinergic hyperactivity may point to seropositivity of MuSK antibodies with therapeutic implications in clinical practice.

**Supplementary Information:**

The online version contains supplementary material available at 10.1007/s13760-025-02811-1.

A 22- year- old female presented with a 6- month history of asymmetrical eyelid ptosis and fluctuated nasal dysarthria and liquid regurgitation. Her past medical history was unremarkable. During sustained gaze fixation the eyelid ptosis was enhanced because of fatigable weakness of the elevator palpebrae superioris muscle. Eyelid twitching was observed at rest and was exacerbated after orbicularis oculi muscle contraction during tight closure of eyes (video). Twichings were bilateral and prominent in the lower eyelids. The patient reported twichings only on the eyelids and no other part of the body. Motor conduction velocities from right median, ulnar and peroneal nerves were normal and no repetitive compound muscle action potentials (R-CMAP) were recorded. Concentric needle electromyogram revealed muscle twitching in orbicularis oculi. Biceps brachii, quadriceps and gastrocnemius muscles on the right side were normal. Fasciculations, fibrillations or myokymia were not shown in any of the above tested muscles. The clinical diagnosis of myasthenia gravis (MG) was set based on fluctuated and fatigable muscle weakness. MG was confirmed with positive titer of muscle specific kinase antibodies (MuSK Abs: 67 nM, positive > 0.03, radioimmunoprecipitation). CT of the mediastinum revealed residual thymic tissue, compatible with patient’s age. She underwent sessions of plasma exchange for rapid amelioration of bulbar symptoms and received prednisolone and a therapeutic cycle of rituximab with resultant pharmacological remission (PR) of MG and great reduction of MuSK Abs (MuSK Abs 13.2 nM, reduction 80.29% at 6 months after rituximab). The eyelid twitchings were persistent during the whole period including PR, while the patient never received acetylcholinesterase inhibitor (AChE -I) treatment (pyridostigmine). Testing for contactin- associated protein-2 Abs (Caspr2 Abs) with immunofluorescence assay (IFA) was negative. No other factor which could cause neuromuscular hyperexcitability (electrolyte disorder, caffeine intake, drug use) was identified. The pathogenetic mechanism of MuSK Abs causing myasthenia is the inhibition of MuSK-low density lipoprotein receptor-related protein 4 (LRP4) binding with resultant dispersal of acetylcholine receptors (AChRs) clustering [1]. Furthermore, MuSK Abs disrupt MuSK-Collagen Q interaction, resulting in deficient binding of AChE at synaptic cleft and impaired breakdown of acetylcholine [2]. In MuSK-MG, muscle twichings are signs of cholinergic hyperactivity due to effect of MuSK Abs at neuromuscular junction and can be observed irrespectively of ACh E-I treatment and clinical status [3, 4]. Awareness of this association has therapeutic implications because when MuSK-MG is suspected, the beginning dose of pyridostigmine should be lower than usual (15 mg instead of 30 mg per dose). In MuSK-MG, pyridostigmine, because of cholinergic hyperactivity, may cause increased oral and bronchial secretions and deterioration of potentially life- threatening bulbar muscle weakness [5].

## Supplementary Information

Below is the link to the electronic supplementary material.

Supplementary material 1 (MOV 35659.4 kb)

## Data Availability

No datasets were generated or analysed during the current study.
